# Extracellular deposition of matrilin-2 controls the timing of the myogenic program during muscle regeneration

**DOI:** 10.1242/jcs.141556

**Published:** 2014-08-01

**Authors:** Ferenc Deák, Lajos Mátés, Éva Korpos, Ágnes Zvara, Tibor Szénási, Mónika Kiricsi, Luca Mendler, Anikó Keller-Pintér, Béla Ózsvári, Hajnalka Juhász, Lydia Sorokin, László Dux, Nicolas Mermod, László G. Puskás, Ibolya Kiss

**Affiliations:** 1 Institute of Biochemistry, Biological Research Centre, Hungarian Academy of Sciences, Szeged, H-6701 Szeged, Hungary; 2 Institute of Genetics, Biological Research Centre, Hungarian Academy of Sciences, Szeged, H-6701 Szeged, Hungary; 3 Institute of Physiological Chemistry and Pathobiochemistry, Muenster University, D-48149 Muenster, Germany; 4 Institute of Biochemistry, Faculty of General Medicine, University of Szeged, H-6720 Szeged, Hungary; 5 Department of Biochemistry and Molecular Biology, Faculty of Natural Sciences and Informatics, University of Szeged, H-6720 Szeged, Hungary; 6 Avidin Ltd., H-6726 Szeged, Hungary; 7 Institute of Biotechnology, University of Lausanne, and Center for Biotechnology of the University of Lausanne and École Polytechnique Fédérale de Lausanne, CH-1015 Lausanne, Switzerland

**Keywords:** Muscle regeneration, Myogenesis, Matn2 shRNA, TGF-β signaling, BMP signaling, NFI, Trf3

## Abstract

Here, we identify a role for the matrilin-2 (Matn2) extracellular matrix protein in controlling the early stages of myogenic differentiation. We observed Matn2 deposition around proliferating, differentiating and fusing myoblasts in culture and during muscle regeneration *in vivo*. Silencing of *Matn2* delayed the expression of the Cdk inhibitor p21 and of the myogenic genes *Nfix*, *MyoD* and *Myog*, explaining the retarded cell cycle exit and myoblast differentiation. Rescue of Matn2 expression restored differentiation and the expression of p21 and of the myogenic genes. TGF-β1 inhibited myogenic differentiation at least in part by repressing *Matn2* expression, which inhibited the onset of a positive-feedback loop whereby Matn2 and Nfix activate the expression of one another and activate myoblast differentiation. *In vivo*, myoblast cell cycle arrest and muscle regeneration was delayed in *Matn2^−/−^* relative to wild-type mice. The expression levels of *Trf3* and myogenic genes were robustly reduced in *Matn2^−/−^* fetal limbs and in differentiating primary myoblast cultures, establishing Matn2 as a key modulator of the regulatory cascade that initiates terminal myogenic differentiation. Our data thus identify Matn2 as a crucial component of a genetic switch that modulates the onset of tissue repair.

## INTRODUCTION

Skeletal muscle regeneration following injury is a multistep process that restores the tissue architecture by the sequential activation of multiple signaling pathways (Chargé and Rudnicki, 2004; Wagers and Conboy, 2005; Yin et al., 2013). The early degenerative phase involves inflammation, necrosis of the injured myofibers and degeneration of motor endplates, leading to denervation. The muscle is then repaired in four regeneration stages. Repair starts with the activation of satellite cells, which reside between the basement membrane and the sarcolemma of muscle fibers, to create a pool of proliferating myoblasts. This is followed by proliferation, differentiation and maturation stages, recapitulating the embryonic differentiation steps.

Muscle differentiation is directed by a conserved myogenic regulatory program during embryonic and fetal development and muscle regeneration (Chargé and Rudnicki, 2004; Shi and Garry, 2006). The proliferating myoblasts express the myogenic regulatory factors (MRFs) Myf5 and MyoD. MyoD initiates a complex muscle-specific gene expression program that also involves a network of other transcription factors committing myogenic precursors to differentiate (Aziz et al., 2010; Yokoyama and Asahara, 2011). This is accompanied by a switch of the core transcription machinery, as the MyoD-dependent activation of the myogenin gene (*Myog*) requires the replacement of TFIID with the TRF3–TAF3 complex (Deato and Tjian, 2007, Deato et al., 2008). The expression of Myog and Mrf4 in committed myoblasts leads to the upregulation of the Cdk inhibitor p21 and to irreversible cell cycle exit (Walsh and Perlman, 1997). Subsequently, overt differentiation starts with the expression of muscle-specific contractile proteins, and the cells either fuse to form primitive multinucleated myotubes or merge with existing myofibers.

The myogenic program is fine-tuned during embryonic and fetal development and during muscle regeneration by morphogenetic regulation in response to inductive signals of growth factors (e.g. FGF, IGF and TGF-β), cytokines and various stimuli from the extracellular matrix (ECM) (Chargé and Rudnicki, 2004; Wagers and Conboy, 2005). TGF-β family members inhibit muscle differentiation and growth following receptor binding and the initiation of specific intracellular signals by Smad2 and Smad3 proteins, whereas BMP signaling through Smad1, Smad5 and Smad8 is a positive regulator of muscle growth (Gardner et al., 2011; Sartori et al., 2009; Sartori et al., 2013; Winbanks et al., 2013). Recent data have also implicated specific members of the nuclear factor I (NFI/Nfi) family of transcription factors in muscle morphogenesis (Gronostajski, 2000), Nfia being responsible for the induction of embryonic muscle genes, whereas Nfix activates fetal genes (Messina et al., 2010).

The ECM has a variety of direct and indirect effects on muscle differentiation and regeneration. Importantly, the inhibition of ECM assembly interferes with myogenic differentiation independently of MRF expression (Osses and Brandan, 2002). During myoblast differentiation, the fibronectin-rich ECM is replaced by a laminin-211-containing basement membrane deposited by myotubes, which influences myogenic cell migration and differentiation, myofiber formation and innervation (Gullberg et al., 1998; Thorsteinsdóttir et al., 2011). A structurally and functionally specialized ECM mediates nerve–muscle and muscle–tendon contacts at neuromuscular junctions (NMJ) and myotendinous junctions (MTJ), respectively (Sanes, 2003). Although ample evidence revealed a significant role for ECM components and cell–ECM interactions at all stages of muscle development (Thorsteinsdóttir et al., 2011), how distinct ECM components might contribute to skeletal muscle differentiation and regeneration remains largely unknown.

Microarray analysis has shown an increased expression of several ECM genes in a mouse model of muscle regeneration, including the matrilin-2 gene (*Matn2*) (Goetsch et al., 2003). Matn2 is the largest member of the matrilin family of multidomain adaptor proteins (Deák et al., 1997; Mátés et al., 2002). Matrilin oligomers interact with other ECM proteins to form filamentous networks by connecting collagen fibrils and proteoglycans (Deák et al., 1999; Klatt et al., 2011). Matn2 homo-oligomers are deposited in varying amounts in the uterus, heart, skeletal and smooth muscle, in loose and dense connective tissues, and they are associated with subepithelial basement membranes (Piecha et al., 1999). Recent studies have demonstrated that Matn2 is required for peripheral nerve regeneration (Malin et al., 2009), and that *Matn2* regulation by the BMP7–Smad signaling pathway can modulate skin-wound healing (Ichikawa et al., 2008).

Here, we reveal transient *Matn2* upregulation during notexin-induced muscle regeneration *in vivo* and in differentiating myoblast cultures. We report delayed myogenic differentiation from *Matn2*-silenced myoblast cultures, and a mild dystrophy with delayed muscle regeneration in *Matn2^−/−^* mice. We also establish that TGF-β can inhibit C2 myoblast differentiation by decreasing *Matn2* expression, and lack of Matn2 delays the onset of *Trf3*, *Nfix* and MRF gene expression. Overall, our data implicate Matn2 in a signaling network controlling the onset of the myogenic program, thereby linking ECM–cell interactions to tissue differentiation and repair.

## RESULTS

### Transient *Matn2* upregulation during skeletal muscle regeneration

**Fig. 1.**
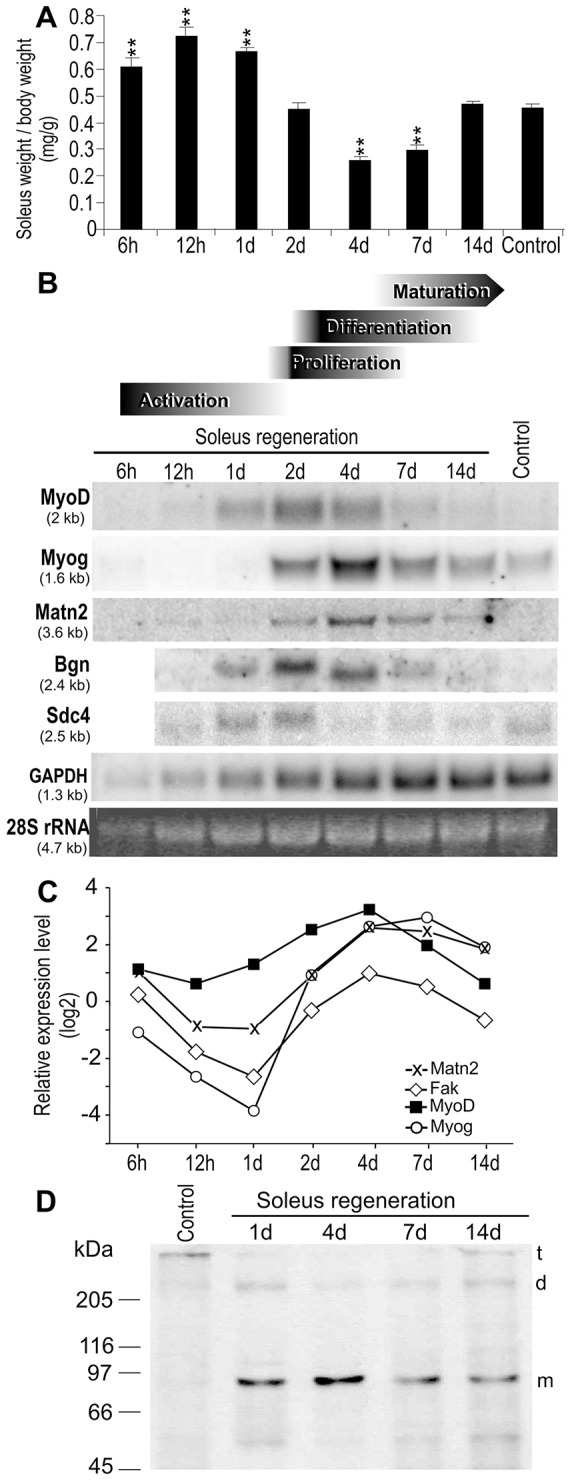
**Marker gene expression during rat soleus regeneration.** (A) Weight changes during notexin-induced regeneration. Data show the mean±s.e.m. (*n*≧3); **P<0.01. (B,C) Kinetic changes in marker gene expression were aligned with the stage of regeneration (Shi and Garry, 2006), based on histological analysis of H&E-stained sections (Fig. 2A). Pooled total RNA derived from control and regenerating samples (*n* = 3) was subjected to northern hybridization (B) and QRT-PCR using the SYBR green protocol (C). For QRT-PCR, relative expression levels normalized to those of the untreated control (ΔΔC_t_) are shown. (D) Immunoblot analysis of Matn2 expression during regeneration. t, trimer; d, dimer; m, monomer.

We first assessed a potential role for Matn2 in muscle regeneration using the well-established model of notexin-induced rat soleus regeneration. Changes in the injected soleus weight over time reflected the expected progress of inflammation, necrosis and repair processes (Fig. 1A). Northern analysis showed marked *Matn2* upregulation until 4 days after treatment, followed by a decline in its expression (Fig. 1B). The transient mRNA accumulation partially overlapped the expression profiles of the MyoD and Myog myogenic differentiation markers, but differed from the induction kinetics of other ECM components, such as biglycan (Bgn) and syndecan-4 (Sdc4). Quantitative RT-PCR (QRT-PCR) analysis of *Matn2* mRNA revealed a 6.5-fold transient activation that followed the peak of expression of *MyoD* and closely paralleled that of *Myog* (Fig. 1C). The *Matn2* mRNA level increased similarly to that of Fak (also known as Ptk2), a common integrin target (Goody and Henry, 2010). We concluded that *Matn2* is upregulated during the regeneration phase, which involves myoblast proliferation and differentiation to myocytes, early myotubes and myofibers, whereas it is downregulated during myofiber maturation.

Immunoblots revealed predominantly Matn2 oligomers of low signal intensity in the untreated control, whereas mainly Matn2 monomers were observed during regeneration. The elevated monomer signal at 1 day post-injury likely resulted from the proteolytic cleavage of oligomers that might be poorly recognized by the antibody in the control (Fig. 1D). The amount of monomers decreased after day 4, whereas the oligomer formation was restored.

### Immunolocalization of Matn2 in regenerating muscles

**Fig. 2.**
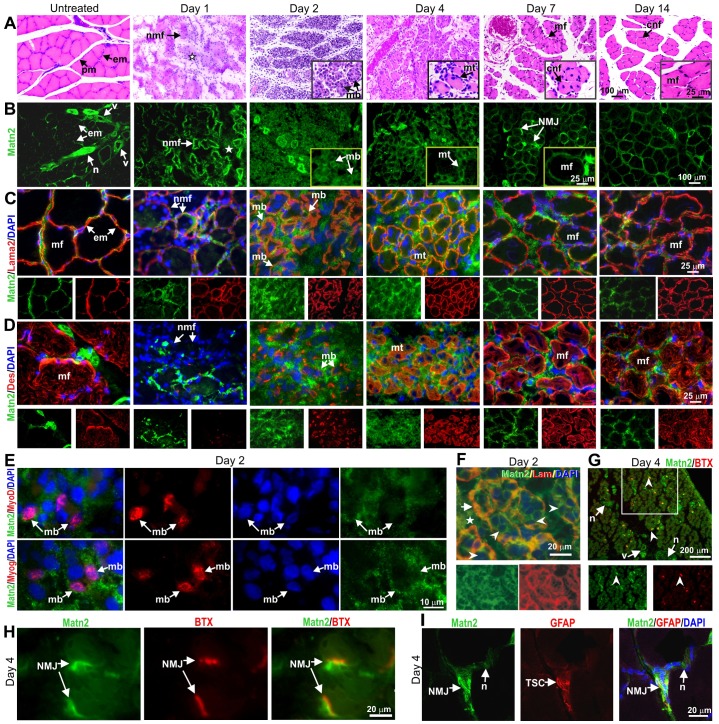
**Progress of morphological changes and Matn2 deposition during rat soleus regeneration.** (A) H&E staining of transverse paraffin sections. The star indicates inflammatory cells. (B–D) Immunofluorescence of serial cryosections for Matn2 (B–D) and laminin α2 (Lama2) (C) or desmin (Des) (D). The star indicates interstitial tissue (B). (E) Deposition of Matn2 around MyoD- and Myog-expressing proliferating and differentiating myoblasts at day 2 post-injury. (F) Matn2 deposition in the interstitial tissue (star) and around myoblasts (arrowhead) differentiating and fusing within the basement membrane, which is also stained for laminin-111 (Lam, arrow), at 2 days post-injury. (G–I) Localization of Matn2 to NMJs on day 4. Note the strong Matn2 signal at the newly established NMJs, as visualized by Rhodamine-labeled α-bungarotoxin (BTX) staining of acetylcholine receptors (G, arrowhead). There is partial overlap between Matn2 and α-bungarotoxin signals at NMJs (H, arrows). TSCs visualized by GFAP staining are covered by Matn2-rich ECM at the NMJ (I). Insets show a section of the main image at higher magnification (A,B). Single-channel images of the entire image (C,D,F) or the outlined area (G) are shown beneath the main image. cnf, centrally nucleated myofiber; em, endomysium; mb, myoblast; mf, myofiber; mt, myotube; n, nerve; nmf, necrotic myofiber; pm, perimysium; v, blood vessel.

To reveal which cell types might express *Matn2*, we compared the localization of Matn2 with that of specific markers during known stages of muscle regeneration (Chargé and Rudnicki, 2004; Gorbe et al., 2005; Shi and Garry, 2006), which were determined by hematoxylin and eosin (H&E) staining of muscle sections (Fig. 2; supplementary material Fig. S1). Unlike the weak Matn2 staining of the endomysium relative to nerves and blood vessels in the untreated muscle, an intense Matn2 signal was observed at the periphery of necrotic myofibers and in the interstitial tissue at day 1 post-injury (Fig. 2A–D; supplementary material Fig. S1A,D). This further implied a proteolytic release of Matn2 epitopes from oligomers and ECM linkages in the old endomysium, thereby resulting in better recognition by the antibody. Using myogenic, basement membrane and inflammatory cell markers, we observed Matn2 deposition around MyoD-, Myog- and Lama2-positive proliferating and differentiating myoblasts on day 2, but not around granulocytes and macrophages (Fig. 2B–F; supplementary material Fig. S1B,C; data not shown). Matn2 colocalized with laminin in the basement membrane surrounding desmin-positive fusing myoblasts, myotubes and myoblasts merging with newly formed myofibers (Fig. 2B–D,F), implying that both proteins can participate in the ECM assembly that assists myoblast differentiation and fusion. Matn2 signal was also relatively strong in the endomysium, but not in the laminin-containing basement membrane of recently formed myofibers strongly stained for desmin (Fig. 2B–D) but still containing central nuclei on day 14 (arrow, Fig. 2A). Matn2 expression decreased during myofiber maturation, but it was more intense and broader in the endomysium and interstitial tissue on day 14 than in the control (Fig. 2B–D). The Matn2 signal partially overlapped those of fibronectin and collagen-1 throughout the regeneration process (supplementary material Fig. S1D).

While keeping the muscle nerves intact, notexin treatment destroys their motor endplates. Unlike myoblast proliferation, myofiber maturation depends on nerve signals (Grubb et al., 1991; Sesodia and Cullen, 1991). Matn2 deposition was also highly elevated around nerves and the NMJs that were re-established in large number between the recently formed myofibers and nerve terminals at days 4–7 (Fig. 2B,G). Double staining showed intense Matn2 deposition at re-established NMJs, specifically in close proximity to acetylcholine receptors in the postsynaptic membrane (Fig. 2H). The Matn2 signal extended to the synaptic membrane and the presynaptic area around the cap formed by terminal Schwann cells (TSCs) (Fig. 2I). The observed deposition of Matn2 around myoblasts, myotubes, newly formed myofibers and NMJs suggested a role for Matn2 in the early steps of myogenic differentiation and reinnervation.

### Transient *Matn2* activation in differentiating C2 myoblasts

**Fig. 3.**
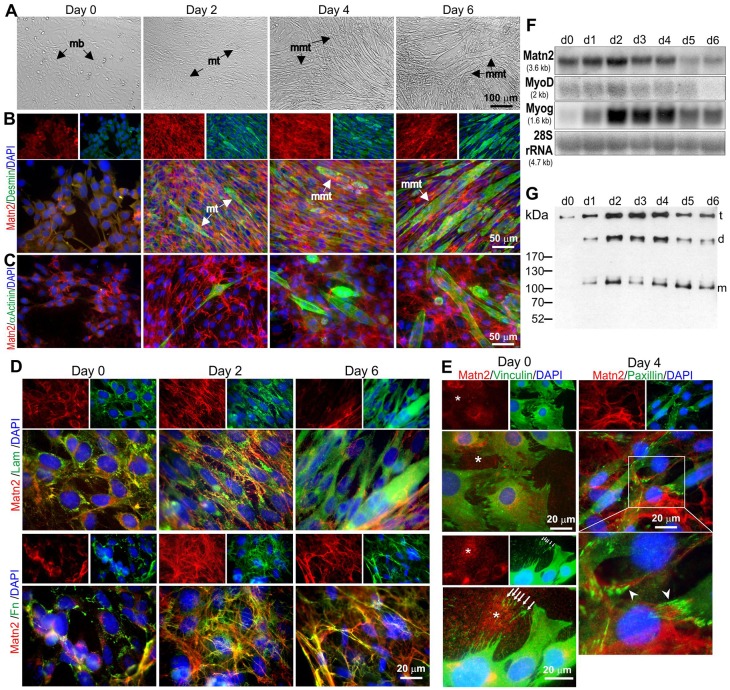
***Matn2* expression in proliferating and differentiating C2 myoblasts.** (A–E) Phase-contrast images (A) and double immunofluorescence for Matn2 and other markers (B–E) of myoblast cultures differentiating to multinucleated myotubes (mmt) in differentiation medium. Desmin (B) and α-actinin staining (C) demonstrates the progress of differentiation. mb, myoblast; mt, myotube. (D) Partial colocalization of Matn2 with laminin-111 (Lam) and fibronectin (Fn). (E) Granular Matn2 deposition (asterisk) by proliferating myoblasts (day 0). Arrows mark the vinculin-positive focal adhesions left behind upon cell movement. Matn2 filaments are linked to the cells at paxillin-positive focal adhesions (arrowhead) on day 4. Separate images for Matn2 alone or for other markers and DAPI are shown above the main images in B, D and E. The area outlined in white in E is shown at higher magnification below the main image. (F) Comparison of mRNA levels for Matn2 and MRFs by northern hybridization. (G) Immunoblot analysis of Matn2 that was secreted daily into the differentiation medium. t, trimer; d, dimer; m, monomer.

To provide independent evidence that myoblasts can deposit a Matn2-rich ECM, we next monitored *Matn2* expression in C2 myoblasts cultured in differentiation medium, as this cell line model was reported to faithfully mimic fetal muscle differentiation (Biressi et al., 2007; Halevy et al., 1995; Messina et al., 2010; Montarras et al., 1996). Polygonal C2 myoblasts proliferating in growth medium (day 0) showed Matn2, laminin and fibronectin deposition, but only traces of desmin and no α-actinin immunofluorescence (Fig. 3A–D). In differentiation medium, myoblasts differentiated to spindle-shaped myocytes and myotubes exhibiting intense desmin, Matn2, laminin and fibronectin signal at day 2, followed by the formation of aligned multinucleated myotube-like structures with high desmin and laminin staining by day 6 (Fig. 3B,D). Long spindle-shaped cells staining for sarcomeric α-actinin appeared in the culture from day 2 (Fig. 3C).

Whereas Matn2 was deposited by proliferating myoblasts in fine granules on day 0, it formed laminin and fibronectin-connected filaments surrounding fusing myoblasts and primary myotubes from day 2 (Fig. 3D,E). The Matn2 filaments appeared to be involved in cell attachment (Fig. 3E), being incorporated into an extensive filamentous network closely associated with fibronectin and connected to the laminin-containing basement membrane around multinucleated myotubes by day 6 (Fig. 3D).

The *Matn2* mRNA level in differentiating myoblasts also exhibited a transient increase with a peak at day 2, thus overlapping the transient accumulation of *MyoD* and *Myog* mRNAs (Fig. 3F). The amount of Matn2 trimers, dimers and monomers secreted into the medium also increased transiently to a maximum on days 2–4 in differentiation medium (Fig. 3G), concomitantly with the appearance of myotubes. These data thus confirmed the deposition of Matn2 by proliferating myoblasts and during the early steps of C2 myoblast differentiation.

### Matn2 is required for C2 myoblast differentiation

**Fig. 4.**
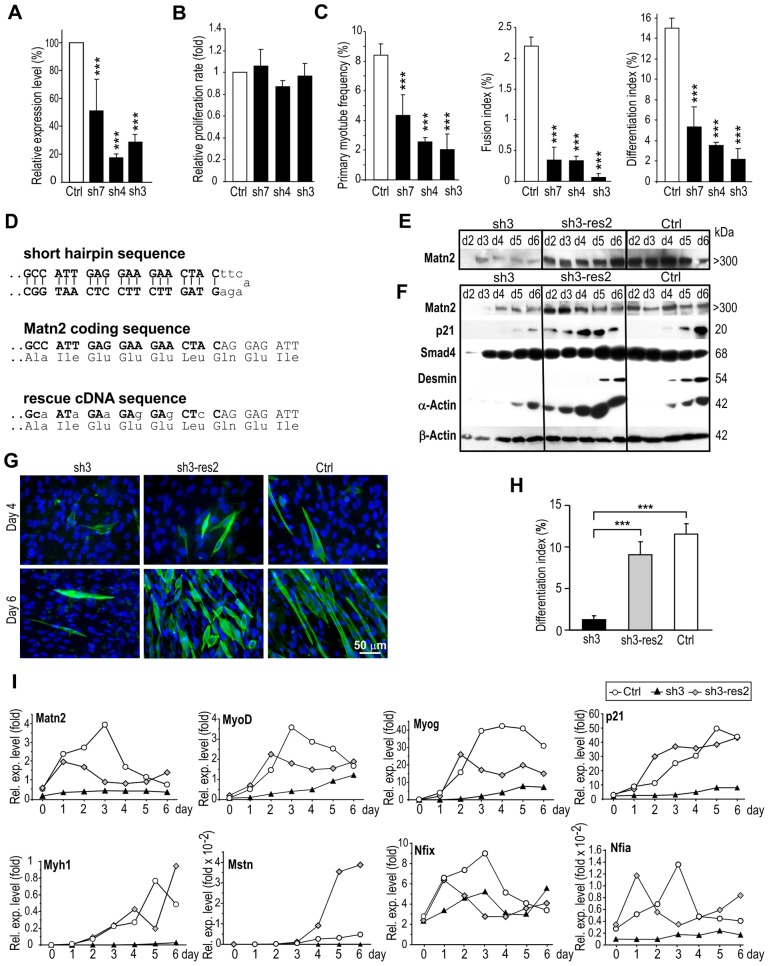
**The effect of *Matn2* silencing and rescue on differentiation and marker gene expression of C2 myoblasts.** (A) *Matn2* mRNA levels were measured by QRT-PCR in proliferating C2 cell lines stably expressing *Matn2* shRNA (sh7, sh4 and sh3) or the control vector (Ctrl). (B) The proliferation rate of the control and silenced cell lines was determined by using the MTS assay in growth medium. (C) Primary myotube frequency, fusion index and differentiation index of the cell lines on day 5 in differentiation medium. (D) Coding sequences are depicted for the *Matn2* shRNA, the endogenous *Matn2* and the rescue cDNA clone, which is resistant to shRNA interference. Immunoblotting was performed on the conditioned differentiation medium (E) and cell lysates (F) from the silenced sh3, rescued sh3-res2 and Ctrl myoblast cultures. Only the trimeric form of Matn2 is shown. Cytoskeletal β-actin served as a loading control. (G) Immunofluorescence for α-actinin-positive cells in the cultures. (H) Differentiation index as determined on day 5. (I) QRT-PCR analysis of relative gene expression (Rel. exp.) in the same cultures using TaqMan probes. Marker mRNA levels are given as fold values relative to the *Hprt* mRNA level. Data show the mean±s.e.m. (A–C, H). ***P<0.001, compared with control (*n* = 3–5). For E–G and I, representative data from three independent experiments are shown.

To address whether Matn2 might be needed for myogenic differentiation, we silenced its expression in C2 myoblasts using an efficient transposon-based short-hairpin (sh)RNA expression system (Kaufman et al., 2005). Independent stable-knockdown lines constitutively expressing either *Matn2*-targeting shRNA (lines sh3, sh4 and sh7) or the control vector (line Ctrl) were established and tested for differentiation and the expression of marker genes. *Matn2* mRNA levels were successfully reduced in lines sh7, sh4 and sh3, with expression levels ranging from 52.2% to 17.6% of that of the control line, as determined by QRT-PCR (Fig. 4A); expression of the Matn2 protein decreased similarly (supplementary material Fig. S2B). Prior to differentiation, the *Matn2*-silenced cells were morphologically identical to control myoblasts (data not shown) and proliferated at similar rates, as based on their mitochondrial activity (Fig. 4B) and on cell count data (data not shown). Upon culturing in differentiation medium, myotube formation was delayed by 2–3 days and the frequency of sarcomeric α-actinin-positive myocytes and their fusion to multinucleated myotubes was highly reduced in *Matn2*-silenced myoblast cultures relative to that of control cultures (supplementary material Fig. S2A,B). The primary myotube frequency, fusion index and differentiation index decreased in correlation with the reduction in Matn2 immunofluorescence and mRNA expression of the cultures (Fig. 4A,C; supplementary material Fig. S2B). Thus, silencing *Matn2* expression delayed both the differentiation and fusion of myoblasts.

Delayed myoblast differentiation was further supported by comparative QRT-PCR, which revealed the most robust changes in myogenic gene expression between the control line and the sh4 and sh3 silenced cell lines in repeated differentiation experiments (supplementary material Fig. S2C; data not shown). Thus, *Matn2* silencing interfered with the transient activation of *MyoD* and *Myog*, as well as with the progressive increase in the p21 mRNA level observed in control myoblasts, readily explaining the delayed commitment, cell cycle exit and differentiation of the **silenced cell lines. *Matn2* silencing also hampered the activation of *Fak*, a gene encoding a modulator of cell–ECM contacts, and the transient rise in the expression of ECM genes (e.g. *Lama2*, *Col6a1* and *Sdc4*), the expression of which paralleled that of *Matn2* in the control line. As no marked alteration was seen for the unrelated control p53 and *Col1a1* mRNAs, this indicated that *Matn2* silencing exerts a specific effect on the expression of MRF and differentiation-related genes.

Next, we tested whether rescuing Matn2 synthesis in a silenced cell line can restore the proper timing of myogenic differentiation. To this end, we mutated the sequence targeted by the shRNA in the mouse *Matn2* cDNA in the third positions of codons, thereby preserving its protein-coding capacity while disrupting the shRNA target site (Fig. 4D). Then, we introduced a constitutive expression vector harboring the modified cDNA into *Matn2-*silenced sh3 myoblasts and selected for pools of puromycin-resistant stable clones (sh3-res2) for further analyses (Fig. 4E–I). The Matn2 levels of the complemented sh3-res2 myoblasts reached those of the control line in both the conditioned differentiation medium and in the filamentous ECM extracts (Fig. 4E,F). As expected, given the use of the constitutive CMV promoter to drive Matn2 expression, a decrease in Matn2 level was not noted in the sh3-res2 culture at day 6 in differentiation medium. During differentiation, α-actinin-positive myotubes appeared at a frequency similar to that of the control line (Fig. 4G,H), and the myoblast proliferation rates were similar (data not shown). Bmp signaling, which controls muscle growth (Sartori et al., 2013; Winbanks et al., 2013), yielded comparable effects, as judged from the similar phosphorylation of Smad1/5/8 (supplementary material Fig. S3B). Smad4 expression was not altered, but large differences were seen in the expression of p21 (Fig. 4F). Unlike its low level in sh3 cells, p21 expression could already be detected at day 2 in the rescued culture, and it increased steadily to reach a level of expression similar to that of the control, but at an earlier time-point. Desmin and α-actin expression was also rescued in the sh3-res2 cultures. As Matn2 silencing also altered the phosphorylation of Fak and p42/44 (also known as Erk1/2) (supplementary material Fig. S3B,C), it is likely to modulate several signaling pathways. Thus, the decreased expression of Matn2 hampered the induction of p21 and overt differentiation, despite the increased expression of the TGF-β signaling intermediate Smad4, and this effect could be reversed by restoring Matn2 expression and its deposition in the ECM. QRT-PCR confirmed the restoration of muscle-specific gene expression in differentiating Matn2-rescued myoblasts (Fig. 4I), and the increase in the *Matn2* level was paralleled by the increased expression of *MyoD* and *Myog* and of the adult myosin heavy chain (*Myh1*) mRNAs.

The finding that Matn2 expression might regulate myoblast differentiation prompted us to assess the regulation of transcription factors that are possibly involved in the onset of *Matn2* expression. Nfi proteins have been implicated in the control of adult stem cells and tissue regeneration in connection with TGF-β signaling (Plasari et al., 2009; Plasari et al., 2010). Nfix has been shown to drive a transcriptional switch from embryonic to fetal myogenesis *in vivo* and in C2 culture by suppressing the effect of Nfia and activating fetal muscle genes (Messina et al., 2010). When monitoring *Nfix* or *Nfia* expression during myogenesis, we observed that *Nfix* and, to lesser degree, *Nfia* were indeed activated in both the differentiating control and Matn2-rescued myoblasts, but were either silent or only expressed at low levels in the silenced cells (Fig. 4I). The *Nfix*, *Nfia* and *Matn2* mRNAs accumulated with similar kinetics in Matn2-expressing cells, and their expression preceded myogenic gene expression. This suggested that Matn2 might control myogenic differentiation by regulating the expression of Nfi proteins. Interestingly, a rise in Matn2 expression above a threshold level was likely needed to turn on the expression of *Nfix* and of the downstream *Myh1* and *Mstn* genes in sh3-res2 cultures (compare Fig. 4I and supplementary material Fig. S3A), implying that Matn2 and Nfix might be part of a genetic switch driving an all-or-none differentiation response.

### TGF-β signaling represses *Matn2* expression and myoblast differentiation

**Fig. 5.**
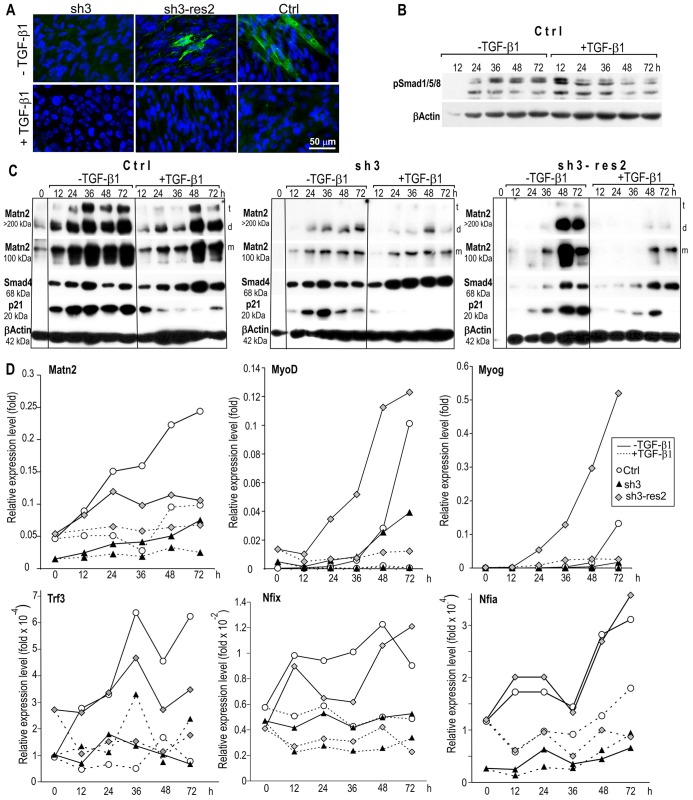
**The effect of TGF-β1 on the differentiation of Ctrl, sh3 and sh3-res2 myoblasts.** Cells were cultured in differentiation medium with and without TGF-β1. (A) Immunofluorescent staining for α-actinin on day 3 of differentiation. (B) Smad1/5/8 phosphorylation during the differentiation of the control cell line was assessed by immunoblotting. (C) Western blot analysis of three pooled samples for Matn2, Smad4 and p21 expression. Cytoskeletal β-actin served as a loading control. t, trimer; d, dimer; m, monomer. (D) QRT-PCR of three pooled parallel cultures using the SYBR green protocol.

*Matn2* has been shown to be regulated by Smad signaling in other cell types (Ichikawa et al., 2008), and TGF-β and Smad2/3 signaling is known to inhibit myogenic differentiation and muscle growth (Gardner et al., 2011; Sartori et al., 2009; Winbanks et al., 2012). Thus, we next assessed whether Matn2 and TGF-β signaling might regulate one another in differentiating myoblasts. As expected, the addition of TGF-β1 to differentiation medium inhibited the differentiation of the control line and hampered Smad1/5/8 phosphorylation (Fig. 5A,B). However, TGF-β1 had only a small delaying effect on the differentiation of sh3 myoblasts, whereas Matn2 expression rescue restored the inhibition (Fig. 5A). TGF-β1 robustly inhibited the increase in *Matn2* mRNA levels, and it consistently reduced the deposition of Matn2 in the ECM from the control and sh3-res2 cultures, especially during the early stages of differentiation (12–36 h, Fig. 5C,D). Similarly, TGF-β1 further reduced the low levels of *Matn2* mRNA and of high-molecular-mass Matn2 complexes from differentiating sh3 myoblasts. Interestingly, the decrease in the p21, *Trf3*, *Nfix* and *Nfia* mRNA levels paralleled the decreased *Matn2* expression, whereas both TGF-β1 and differentiation medium strongly induced Smad4 expression (Fig. 5C,D). The most prominent decrease in p21 expression was obtained in the TGF-β1-treated sh3 cells, which displayed the lowest Matn2 levels and the highest Smad4 levels. As Trf3 is needed for the transition of the basal transcription machinery and for MyoD-dependent Myog induction during myogenesis (Deato and Tjian, 2007; Deato et al., 2008), and both *Matn2* silencing and TGF-β1 compromised *Trf3* induction in differentiation medium, our data suggest that low extracellular Matn2 and TGF-β1 signaling act in concert to inhibit p21 expression and myoblast differentiation. Accordingly, TGF-β1 abolished the induction of *Trf3* and subsequently reduced *MyoD* and *Myog* induction upon differentiation of Matn2-expressing control and sh3-res2 myoblasts, whereas TGF-β1 further reduced the silenced Matn2 level and fully abrogated the expression of the two MRFs from sh3 cells (Fig. 5D). Overall, we concluded that Matn2 can activate *Trf3* and p21 expression and myogenic differentiation, and the TGF-β1–Smad-pathway-mediated inhibition of differentiation might stem in part from the inhibition of *Matn2*, *Trf3*, p21 and perhaps also *Nfix* expression.

### *Matn2* is activated by the myogenic Nfix and MyoD regulators

**Fig. 6.**
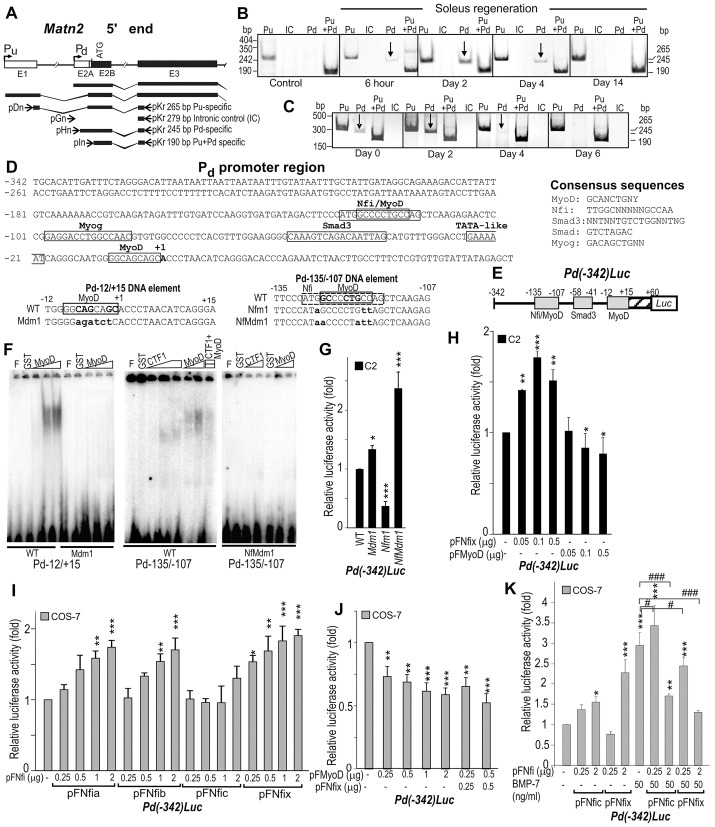
**Regulation of the *Matn2* promoter by MyoD and Nfi.** (A) Schematic illustrating the exons E1–E3, the two alternative promoters and the *Matn2* transcripts. Primer pairs specific for P_u_ and P_d_ and for intronic control (IC) are depicted (arrows), and have been described previously (Mátés et al., 2002). Semi-quantitative RT-PCR analysis using P_u_- and P_d_-specific and control primer pairs during soleus regeneration (B) and C2 myoblast differentiation (C). (D) Nucleotide sequences showing the putative factor-binding sites in the P_d_ region and in the wild-type (WT) and mutant versions of DNA elements Pd−12/+15 and Pd−135/−107. Mutated nucleotides are shown in lower case. (E) Structure of the P_d_-luciferase fusion construct. (F) The binding of purified GST (glutathione S-transferase), GST–MyoD and GST–CTF1 (NFIC isoform) to the wild-type and mutant Pd−12/+15 and Pd−135/−107 DNA elements was determined in EMSA. F, free probe. (G) The effect of the NFI and MyoD motif mutations shown in D on the P_d_ activity of *Pd(−342)Luc* in C2 myoblasts. (H–K) The effect of forced expression of NFI proteins and MyoD from the indicated amounts of expression plasmids (pFNfi and pFMyoD) on the luciferase activity of *Pd(−342)Luc* in C2 myoblasts and COS-7 cells. In COS-7 cells, NFI proteins can increase P_d_ activity (I), whereas MyoD alone or upon coexpression with Nfix can repress it (J). Bmp7 can increase P_d_ activity and its dose-dependent activation by Nfic and Nfix (K). Data represent the mean±s.e.m.; *P<0.05, **P<0.01, ***P<0.001 [relative to *Pd(−342)Luc*], ^#^*P*<0.05, ^###^*P*<0.001 (as indicated).

Based on the observation that *Matn2* and *Nfix* mRNAs accumulated with comparable kinetics during early myogenesis and on the previous finding that Nfix can promote myogenesis, we next assessed whether Nfix might directly regulate *Matn2* expression. As *Matn2* is transcribed from two promoters (Mátés et al., 2002), we tested which of the upstream (P_u_) or downstream promoter (P_d_) can direct the transient gene activation during muscle regeneration and myoblast differentiation (Fig. 6A–C). Transcription from P_u_ did not change markedly in either case. By contrast, the P_d_-specific transcript was reproducibly upregulated between 6 h and 4 days after soleus injury, followed by a decline during myofiber formation (Fig. 6B, arrow). Consistently, transcription from P_d_ increased transiently during the early stages of C2 myoblast differentiation, but ceased during the formation of multinucleated myotubes (Fig. 6C, arrow).

Sequence analysis revealed putative NFI- and MyoD-binding motifs in the P_d_ region, including potentially overlapping binding sites for both proteins (Fig. 6D). Electrophoretic mobility shift assay (EMSA) showed MyoD binding to the Pd−12/+15 DNA element near the transcription start site, and this binding was abolished by mutation in the MyoD motif (Mdm1) (Fig. 6D,F). Both MyoD and the CTF1 NFI isoform bound weakly to the Pd−135/−107 upstream element, and point mutations disrupting both consensus motifs (NfMdm1) abolished this interaction. Low amounts of MyoD inhibited NFI binding, indicating that the two proteins can compete for binding to the distal overlapping sites. The Mdm1 mutation moderately increased the P_d_ activity in C2 myoblasts (Fig. 6E,G). An NFI-contact-point mutation (Nfm1) in the overlapping NFI and MyoD sites was strongly inhibitory, whereas the NfMdm1 mutation that eliminated the binding of both proteins increased the promoter activity. In C2 myoblasts, MyoD expression decreased whereas Nfix expression increased the P_d_-driven luciferase activity (Fig. 6H). Nfix expression in COS-7 cells confirmed that it acts as an activator of the P_d_, as did the Nfia and Nfib species, whereas MyoD acted as a repressor (Fig. 6I,J). Coexpression of MyoD and Nfix did not relieve MyoD-mediated repression of the P_d_, indicating that MyoD can fully block the activation by Nfix, in agreement with their competitive binding in EMSA assays and with MyoD high-affinity binding to a site overlapping *Matn2* transcription initiation site. The Matn2 promoter also harbors putative Smad sites that might mediate the TGF-β1 inhibitory effect (Fig. 6D). As BMP7 was reported to activate *MATN2* and its promoter in other cells (Ichikawa et al., 2008), we also assessed the activation of the *Matn2* promoter by BMP7. We found that BMP7 led to a threefold increase in P_d_ activity and increased its transactivation by low doses of Nfic and Nfix (Fig. 6K).

Overall, we conclude that a transient increase in *Matn2* P_d_ activity results from the upregulation of NFI species in early myogenesis, whereas the expression of MyoD at later stages of differentiation might repress *Matn2* expression. A mechanism involving *Matn2* activation at the onset of the myogenic program, together with the finding that Matn2 and Nfix each activate the expression of the other during early myoblast differentiation (Figs 4–6), explains the transient upregulation of *Matn2* during myoblast differentiation and muscle regeneration. The initial increase in *Matn2* expression at day 1 is expected to lead to an increase in *Nfix* and *Nfia* expression (Fig. 4I), which would further increase Matn2 expression. This positive-feedback regulatory loop would ensure high expression of *Nfix*, *Nfia* and *Matn2* at early differentiation stages, whereas the later increase in MyoD expression would contribute to the downregulation of *Matn2* expression in a negative-feedback loop during terminal myogenic differentiation.

### Matn2 deficiency impairs proper muscle regeneration *in vivo*

**Fig. 7.**
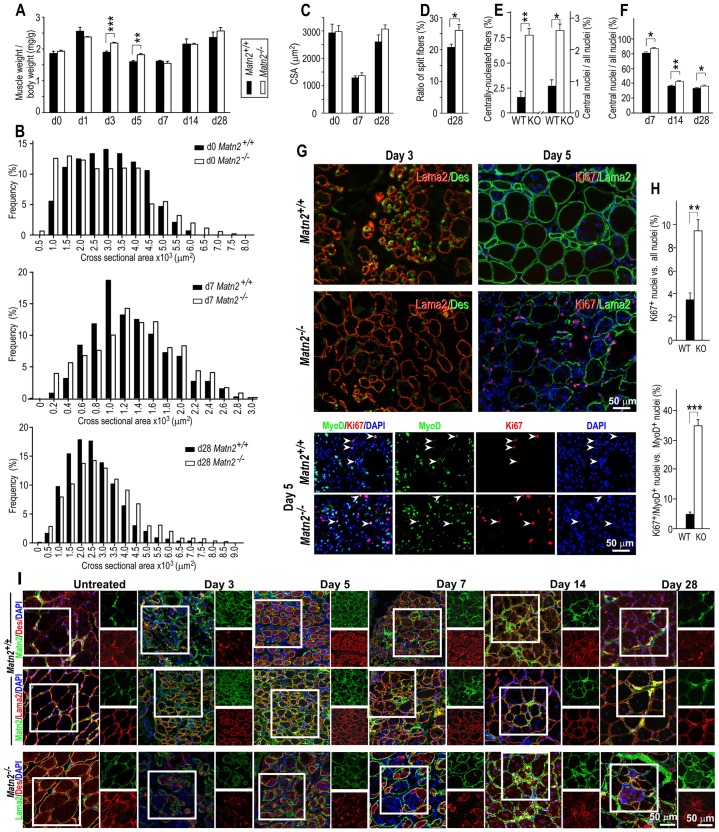
**Mild muscular dystrophy and delayed tibialis anterior muscle regeneration in *Matn2^−/−^* mice.** (A–F) Tibialis anterior regeneration was quantified at the indicated days after notexin injection in wild-type (WT, closed bars) and *Matn2^−/−^* (KO, open bars) mice. (A) Weight changes during regeneration (*n* = 3–6). The CSA frequency distribution (B) and average CSA (C) (300–400 myofibers/muscle, *n* = 3). (D) Quantification of split myofibers on day 28 post-injury. The ratio of centrally nucleated versus all myofibers of untreated tibialis anterior (E, left) and the ratio of non-peripheral versus all myonuclei of untreated (E, right) and regenerating (F) adult tibialis anterior are shown (2000 myonuclei/animal, *n* = 3). (G) Prolonged proliferation in Matn2-deficient regenerating muscles was revealed by Lama2/desmin, Ki67/Lama2 and Ki67/MyoD immunofluorescence. Arrowheads point to Ki67^+^MyoD^+^ cells. (H) Quantification of Ki67^+^ versus all nuclei and Ki67^+^MyoD^+^ nuclei within the MyoD^+^ nuclei population (10,000–30,000 nuclei/animal group, *n* = 3). (I) Double immunofluorescence indicates transient Matn2 upregulation between days 3–7 around desmin-positive myoblasts and myotubes. In Matn2-deficient mice, the numerous myoblasts still detected inside the basement membrane of regenerating myofibers on days 5 and 7 post-injury indicate delayed regeneration, whereas the enrichment of degenerating and regenerating myofibers on days 14 and 28 shows mild dystrophy. The outlined area is shown as single-channel images to the right of the main image. All quantitative data represent the mean±s.e.m.; *P<0.05, **P<0.01, *** *P*<0.01.

**Fig. 8.**
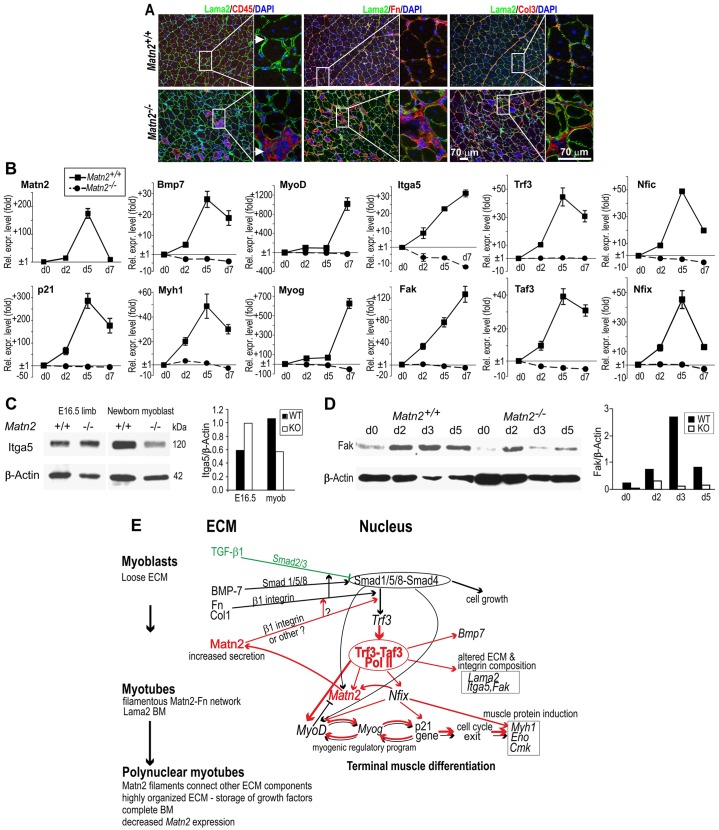
**The effect of Matn2 on marker gene expression during myoblast differentiation.** (A) Impaired regeneration at day 28 in *Matn2^−/−^* mice. Necrotic myofibers accumulate, which attracts numerous CD45-positive inflammatory cells (arrow), leading to increased fibronectin and collagen-3 deposition (magnified insets). (B) QRT-PCR analysis of marker gene expression in *ex vivo* differentiating myoblast cultures derived from *Matn2^−/−^* and *Matn2^+/+^* newborn mice, performed using the SybrGreen protocol. Results show the mean±s.e.m. (C) Immunoblot analysis of integrin α5 in limbs of E16.5 embryos and in wild-type (WT) and *Matn2^−/−^* (KO) primary myoblast cultures. (D) FAK detection by immunoblotting in myoblast cultures differentiating *ex vivo*. For C and D, the proteins were quantified by densitometry. (E) A model of myoblast differentiation. Regulatory interactions linking Matn2 expression to myoblast differentiation are illustrated schematically. Red arrows indicate activation processes affected directly or indirectly by Matn2 signaling. BM, basement membrane; Fn, fibronectin.

Similarly to rat muscles (Fig. 2A,G), high Matn2 signal was observed in nerves, tendons and MTJs, and the signal intensity in the epi-, peri- and endomysium was lower in adults than newborn mice (supplementary material Fig. S4A–G). To further establish the function of Matn2 in myogenesis *in vivo*, we next compared the regeneration of tibialis anterior muscle following notexin-induced injury in *Matn2* knockout (*Matn2^−/−^*) and wild-type mice (Fig. 7). Significant differences were seen in the muscle weight to body weight ratio at days 3–5 (Fig. 7A). The average myofiber cross-sectional area (CSA) did not differ significantly, but the myofiber size was more heterogeneous and fiber splitting occurred more frequently in *Matn2^−/−^* than in wild-type mice (Fig. 7B–D). The incidence of centrally nucleated myofibers increased fivefold in the untreated *Matn2^−/−^* muscles, and the proportion of central myonuclei was also significantly higher in both the untreated and regenerating muscles of *Matn2^−/−^* mice than in those of wild-type mice, indicating a mild muscular dystrophy (Fig. 7E,F; supplementary material Fig. S4H). Consistently, decreased desmin staining and an increased number of Ki67^+^ and Ki67^+^MyoD^+^ cells were observed in Matn2-deficient mice at days 3 and 5 of regeneration, respectively (Fig. 7G,H). Immunofluorescence revealed a delayed regeneration in *Matn2*-deficient mice, as indicated by the delayed desmin staining and the large number of myoblasts still detected inside the basement membrane of the necrotized and regenerating *Matn2^−/−^* muscle fibers at days 5 and 7 post-injury (Fig. 7I). At late stages of regeneration, signs of mild muscular dystrophy were seen in *Matn2^−/−^* mice, including necrosis, inflammation and increased fibrosis (Fig. 7I; Fig. 8A; supplementary material Fig. S4H). Central myonuclei and myofiber splitting also occurred more frequently, whereas fibrosis was not more pronounced in older *Matn2^−/−^* than in wild-type animals (supplementary material Fig. S4I,J).

Next, we tested the effect of Matn2 deficiency on the muscle-specific gene expression in fetal limbs and myoblasts derived from newborn mice. Remarkably, QRT-PCR analysis revealed a 361-fold drop in *Trf3* expression, followed by robust downregulation in the expression of *Nfix*, *Nfia*, *Bmp7* and late differentiation markers (*Eno*, *Myh1*) in *Matn2^−/−^* fetal limbs at embryonic day 16.5 (E16.5) (Table 1). Matn2 deficiency also significantly decreased the expression of the *MyoD*, *Fak* and *Taf3* myogenesis regulators. Because an Nfix-directed switch from embryonic to fetal myogenesis takes place between E14.5 and E17.5 (Messina et al., 2010), along with the switch of the TFIID to a Trf3–Taf3-containing complex (Deato and Tjian, 2007), our data imply that Matn2 deficiency impairs the global switch towards terminal muscle differentiation by robustly preventing the induction of myogenic regulators, such as *Trf3* and *Nfix*.

**Table 1.**
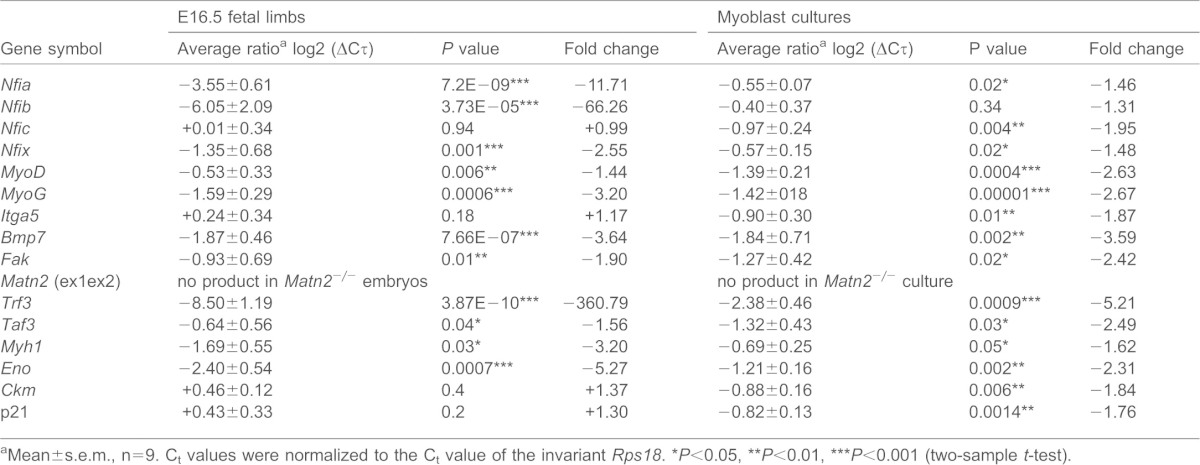
Comparison of the expression levels of marker genes in fetal limbs and myoblast cultures from *Matn2^−/−^* versus wild-type mice by QRT-PCR

a Mean±s.e.m., n = 9. C_t_ values were normalized to the C_t_ value of the invariant *Rps18*.

* *P*<0.05,

** *P*<0.01,

*** *P*<0.001 (two-sample *t*-test).

The regulatory cascade induced by Matn2 is elicited by the myoblasts themselves, as the expression of *Trf3*, *Taf3*, *Bmp7*, *MyoD*, *Fak*, *Nfic*, *Nfix* and *Itga5* were all decreased in *Matn2^−/−^* versus *Matn2^+/+^* cultured myoblasts (Table 1). When the kinetics of primary myoblast differentiation were assessed in differentiation medium, a transient 160-fold activation of *Matn2* expression was accompanied by a 28–50-fold transient induction of *Tfr3*, *Taf3*, *Nfix*, *Nfic*, *Nfia* and *Bmp7* and by elevated expression of *Itga* and *Fak* in differentiating *Matn2^+/+^* myoblasts (Fig. 8B). Interestingly, after a 50–100-fold activation on day 2, the levels of *MyoD* and *Myog* peaked at day 7, following the earlier activation of p21 and *Myh1* with a kinetics similar to those of *Trf3*, *Taf3* and *Nfi*.

By contrast, in the absence of Matn2, the expression of *Trf3*, *Taf3*, *MyoD*, *Myog* and other myogenic marker genes was not induced (Fig. 8B). Thus, Matn2 deficiency interfered with the transient activation of *Nfix* and *Nfia* regulating fetal and embryonic muscle genes, and it also prevented the induction of the *Bmp7* and *Nfic* intermediates of the BMP/TGF-β signaling pathways. Decreased Itga5 and Fak expression in *Matn2^−/−^* myoblasts and during their differentiation, respectively, indicated that Matn2 deficiency can also affect integrin signaling (Fig. 8C,D). p42 (Erk2) phosphorylation was also inhibited in *Matn2^−/−^* mice (supplementary material Fig. S3C).

Taken together, our data indicate that the myoblast-secreted Matn2 is needed for *Trf3* and *Taf3* induction during differentiation, thereby selectively turning on the cascade of MRFs and Nfix, the activator of fetal muscle genes. Thus, we conclude that Matn2 acts as a key and early regulator of fetal myogenic differentiation, and that its transient activation and the feedback regulatory loops it forms with Nfix and MyoD might drive proper and timely BMP7 and integrin α5 signaling during the early stages of muscle differentiation.

## DISCUSSION

Numerous studies of normal and myopathic muscles have underlined the crucial dependence of muscle development and function upon proper cell–ECM interactions. However, how this interaction can contribute to myogenic differentiation has remained poorly understood. This study uncovers a crucial role for the ECM component Matn2 in skeletal muscle differentiation and regeneration. We find that a transient upregulation of *Matn2* followed by Matn2 deposition around proliferating, differentiating and fusing myoblasts is required for timely myoblast differentiation in culture and during muscle regeneration *in vivo*. This conclusion is supported by the finding that silencing or knockout of *Matn2* delays myogenic differentiation from cultured myoblasts by inhibiting the activation of MRFs, p21, *Nfix* and other myogenesis-related genes. Conversely, rescuing Matn2 expression in *Matn2*-silenced myoblasts attenuated the delay in differentiation and restored the induction of *Nfix* and myogenic marker genes.

The muscle regeneration delay in *Matn2^−/−^* mice was accompanied by a lack of *Trf3* and *Taf3* induction *in vivo*, thereby compromising the global transition of the core promoter complex and the activation of MRF (*MyoD*, *Myog*), p21 and muscle-specific genes (*Myh1*). In addition to identifying Matn2 as an upstream global modulator of myogenic differentiation, these data further support the proposal that the Trf3–Taf3 complex plays a pivotal role in terminal muscle differentiation by specifically activating a subset of myogenic marker genes while inhibiting many others (Deato and Tjian, 2007; Deato et al., 2008; Jones, 2007). Matn2 deficiency also interferes with the activation of *Nfix*, which defines the fetal myogenic cell fate (Messina et al., 2010). This correlates well with the previous *in vivo* observation of *Matn2* fetal-specific expression during the myoblast–myotube transition (Blais et al., 2005; Biressi et al., 2007; Mourikis et al., 2012). Matn2 might thus induce muscle-specific gene expression changes by coordinating various signaling pathways, as Matn2 deficiency also impairs the activation of genes involved in the Bmp7 and integrin α5 signaling pathways during differentiation.

Based on the present work and data available from the literature, we propose a hypothesis for the Matn2-dependent timely onset of myogenic differentiation (Fig. 8E). Our data show that Matn2 can modify integrin and other (e.g. Fak, Erk2) signaling pathways and that *Matn2* deficiency dramatically impairs the activation of *Trf3* expression and markedly delays the muscle differentiation program. Thus, the Matn2-elicited ECM signaling is needed to turn on *Trf3*, possibly by modifying the composition of multiprotein Smad complexes formed with other transcription factors. As the Trf3–Taf3 complex is known to activate *MyoD* and to cause a global switch towards terminal differentiation (Deato and Tjian, 2007; Deato et al., 2008), this complex might mediate the Matn2-dependent upregulation of MRF and muscle-specific genes in myoblasts (Fig. 8E).

Whereas the loose ECM surrounding myoblasts favors growth factor signaling, the highly organized ECM of polynucleated myotubes and myofibers rather provides a reservoir, releasing growth factors upon damage (Fig. 8E). Matn2 might contribute to cell adhesion by its interaction with integrins and ECM proteins, such as collagens, fibrillins, laminin–nidogen complex and fibronectin (Piecha et al., 2002; Mann et al., 2007; Klatt et al., 2011). Working as an adaptor protein in the ECM, Matn2 might directly or indirectly modulate TGF-β/BMP/Smad and other signaling pathways through its interaction with fibronectin and fibrillin. For instance, TGF-β family members are stored in latent fibronectin- or fibrillin-bound structures in the ECM, and their activation requires integrin binding and contractile forces (Massagué, 2012). Matn2 deficiency also impaired p42 (Erk2) phosphorylation that was implicated in myoblast fusion (Knight and Kothary, 2011). However, further studies are required to fully understand how the extracellular Matn2-elicited signals are transduced to the cell nucleus.

Our model is in line with previous observations that TGF-β1 acts to inhibit myoblast differentiation (Schabort et al., 2009). It also incorporates the finding that TGF-β1 opposes BMP signaling and *Matn2* expression, thus preventing the onset of a positive-feedback loop whereby Matn2 and Nfix activate the expression of one another (Fig. 8E). This positive-feedback loop might ensure that sufficient levels of both Matn2 and Nfix are reached, such that Nfix can, in turn, promote the initiation of the muscle-specific gene expression program driven by further positive autoregulatory loops formed by MyoD, Myog and p21 (Halevy et al., 1995; Walsh and Perlman, 1997; Aziz et al., 2010). This regulatory cascade fits well with prior observations of the concomitant induction of MRF genes (*MyoD*, *Myog*, *Mef2a*) and fetal muscle genes (*Myh7*) by forced *Nfix* expression in embryonic myotubes and in differentiating C2 cells (Biressi et al., 2007; Messina et al., 2010).

By controlling the expression of regulatory and ECM target genes, Nfix can increase MyoD-dependent muscle-specific gene expression (Aziz et al., 2010). Whether Nfix can directly control the *MyoD*, *Myog* and/or p21 genes, and whether a feedback loop also occurs between these regulatory intermediates, remains to be established. However, this would be consistent with prior reports of Nfi-binding sites in the *Myog* and p21 promoters (Johanson et al., 1999; Ouellet et al., 2006), and with the finding that Nfi species can act as a cofactor in muscle-specific transcription (Funk and Wright, 1992; Messina et al., 2010). Our interpretation of a regulatory interplay between Nfix and cell cycle inhibitors, such as p21, is also supported by a previously described role of Nfix in the regulation of mitogenic pathways in other cell types (Nebl et al., 1994). Our conclusions are also in line with prior observations of regulatory relationships between TGF-β and other Nfi species (Plasari et al., 2009; Plasari et al., 2010) and of BMP7 and Matn2 (Ichikawa et al., 2008) to control the timing of skin-wound healing. In keeping with these findings, we observed severe wound-healing defects in *Matn2^−/−^* mice on a C57BL/6 genetic background (F. Deák and I. Kiss, data not shown). Our results provide evidence for a molecular mechanism whereby TGF-β1-mediated regulation of *Matn2* might concomitantly control NFI expression.

Finally, our model provides a mechanistic explanation for the transient activation of *Matn2*, *MyoD* and *Myog* during myogenic differentiation. As *MyoD* expression is turned on in response to increased expression of Matn2 and possibly also of Nfix, a negative-feedback loop can be established whereby MyoD, overcoming the effect of Nfix, will block *Matn2* expression at late stages of differentiation (Fig. 8E). This is accompanied by the concomitant repression of *Matn2*, *Nfix* and *MyoD*, whereas the expression of MyoD targets and downstream regulators of myogenesis, such as Myog, persists until later stages, when components of terminally differentiated myofibers, such as Myh1, become expressed. The involvement of MRFs in Matn2 regulation is supported by recent online reports of chromatin immunoprecipitation data from the ENCODE consortium that reveal a possible binding of MyoD to the *Matn2* promoter in C2 myoblasts and increased promoter occupancy by Myog in C2 myocytes.

In the absence of Matn2, BMP signaling alone or in combination with signaling by other ECM proteins might also activate MyoD and the myogenic regulatory program, but less efficiently and more slowly than upon *Trf3* induction in the presence of Matn2 (Fig. 8E, black arrows). This can explain why *Matn2* deficiency only delays myogenic differentiation, causing mild dystrophy, but does not block muscle differentiation or regeneration in transgenic mice, as *Matn2^−/−^* mice develop without obvious abnormalities (Mátés et al., 2004). No deficiency in embryonic or fetal muscle development was observed by light microscopy. However, we recently observed that *Matn2^−/−^* mice display an increased susceptibility to diethylnitrosamine-induced hepatocarcinogenesis (Fullár et al., 2014) and wound-healing defects with variable severity depending on the genetic background (F. Deák and I. Kiss, data not shown). Muscles of *Smad^−/−^* mice also showed a mild myopathy, but denervation caused a dramatic atrophy (Sartori et al., 2013).

Overall, this work identifies Matn2 as an important ECM protein required for proper and timely myogenic differentiation and skeletal muscle regeneration. As TSCs induce and guide the sprouting of nerves at NMJs (Son et al., 1996), the Matn2-rich matrix produced by TSCs at NMJs possibly also facilitates the reinnervation process. Matn2 likely contributes to a broad range of repair processes, as it is expressed in a variety of committed proliferating cell types and it functions in regenerative processes of the liver (Szabó et al., 2007), skin (Ichikawa et al., 2008) and peripheral nerves (Malin et al., 2009). These data together support the hypothesis that Matn2 controls ECM–cell communication, and that it can thereby regulate the early steps of cell differentiation and repair processes of various tissues, in concert with NFI transcription factors and TGF-β signaling pathways. Accordingly, other members of the NFI family have been implicated in other regenerative processes of adult tissue in the context of TGF-β signaling. For instance, Nfic has been implicated in tooth morphogenesis, skin-wound healing and the onset of the follicle cycle leading to hair regeneration (Lee et al., 2009; Plasari et al., 2009; Plasari et al., 2010). An NFIC isoform was shown to harbor a TGF-β-responsive domain, and NFI-binding sites acting as TGF-β-responsive elements have been reported in several other ECM genes (Alevizopoulos et al., 1995; Alonso et al., 1996; Iozzo et al., 1997). Collectively, these findings link the NFI transcription factors and ECM target proteins, such as Matn2, to the regulation of the onset of a variety of adult tissue regeneration processes.

## MATERIALS AND METHODS

### Animal treatment

For this study, 3-month-old Wistar rats were anesthetized by intraperitoneal injection of 4% chloral hydrate (10 µl/g). Muscle necrosis was induced by intramuscular injection of 20 µg of notexin, as described previously (Zádor et al., 1996). The animals were killed by an overdose of sodium pentobarbital. The entire soleus muscles of control or notexin-treated legs were removed, weighed and stored at −80°C after freezing in isopentane cooled by liquid nitrogen.

The generation of *Matn2^−/−^* mice has been reported previously (Mátés et al., 2004). Muscle regeneration was induced in 10-week-old *Matn2^−/−^* mice and wild-type littermates by injecting 0.3 µg (60 µl) of notexin (Sigma-Aldrich) into the tibialis anterior muscle. All animal treatments were conducted in accordance with the National Institutes of Health Guide for the Care and Use of Laboratory Animals and under the approval of the Animal Health Care and Control Institute, Csongrád County, Hungary.

### Cell culture

C2 myoblast cell line clone 7 (Yaffe and Saxel, 1977; Montarras et al., 1996) and primary myoblasts, which were prepared from the thigh muscle of neonatal mice as described previously (Springer et al., 1997), were propagated in growth medium containing 20% fetal bovine serum (FBS, Gibco) (Halevy et al., 1995). Differentiation was induced by switching the 80% confluent cultures to differentiation medium containing 2% horse serum, and this was defined as day 0. The cultures were photographed with an Olympus Cell R microscope. In some experiments, 5 ng/ml TGF-β1 (Immunotools) was added to differentiation medium on day 0 and was changed daily. Differentiation experiments were repeated at least three times.

### Histology, immunohistochemistry and immunofluorescence

The average CSA and fiber-size distributions were determined using the Digimizer software (MedCalc Software, Mariakerke, Belgium). Immunohistochemistry was performed as described previously (Piecha et al., 1999). Primary and secondary antibodies used for immunofluorescence of acetone-fixed 10-µm cryosections and cells grown on coverslips are listed in supplementary material Table S1. Nonspecific binding of the antibodies was blocked with 10% proper normal serum. Nuclei were stained with DAPI. NMJs were detected with tetramethylrhodamine α-bungarotoxin (Invitrogen, 1∶200). The specimens were mounted with fluorescent mounting medium (Dako), viewed with a Nikon Eclipse E600 microscope equipped with epifluorescence and photographed with a Spot RT Slider camera. The images were processed using SPOT software (version 4.0.9 for Windows; Diagnostic Instruments). Figures were created with Adobe Photoshop 8.0 and CorelDraw X4 softwares.

### Protein extraction and immunoblot analysis

The myoblast cell layer was extracted in ice-cold buffer containing 20 mM Tris-HCl pH 7.5, 0.15 M NaCl, 1 mM EDTA, 1 mM EGTA, 1% Triton X-100 and protease inhibitors. After centrifugation at 10,000 ***g*** for 10 min at 4°C, supernatant proteins were separated by SDS-PAGE, blotted and developed as described previously (Piecha et al., 1999). Primary and HRP-conjugated secondary antisera are listed in supplementary material Table S1. Images were quantified with the Quantity One-4.2.3. program (BioRad). Experiments were repeated at least three times.

### RNA analysis

Pooled total RNA was isolated from rat soleus muscles (*n* = 3) and used for northern analysis as described previously (Deák et al., 1997), using gene-specific cDNA fragments (supplementary material Table S2). For QRT-PCR, pooled total RNA was isolated from three cultures of differentiating C2 cell lines, three primary myoblast cultures grown either in growth medium or differentiating *ex vivo* in differentiation medium and also from E16.5 fetal limbs. QRT-PCR was performed with the SYBR green protocol, as described previously (Nagy et al., 2011). TaqMan probe sets and the TaqMan Gene Expression Master Mix (Applied Biosystems) were used with the following program: 15 min at 95°C; 45 cycles of 95°C for 15 s and 60°C for 1 min. Gene-specific primers and TaqMan probe sets are listed in supplementary material Table S2. Individual threshold cycle (C_t_) values were normalized either to the average C_t_ values of three internal control genes (*Hprt*, *Rps18* and *CycloAb*) (SYBR green) or to the C_t_ values of *Hprt* (TaqMan probe). Relative gene expression levels are presented as log_2_ ratios or fold values.

### Silencing and rescuing *Matn2* expression

We designed shRNA targeting Matn2 using the program RNAi explorer (Fig. 4D). Oligonucleotides were annealed, phosphorylated and inserted into the pFP/Neo-H1 vector (Kaufman et al., 2005), following recommendations for the pSUPER system to generate the derivative KD2639. A total of 1×10^5^ C2 myoblasts were co-transfected in growth medium with 500 ng of purified KD2639 or pFP/Neo-H1 control DNA and 50 ng of pFvFP helper vector using ExGene 500 (Fermentas). At 24 h after plating, the cells were seeded into 10-cm dishes for a 2-week selection in growth medium containing 500 µg/ml G418 (Cambrex), and stably transfected *Matn2*-silenced cell lines were established from G418-resistant single colonies.

To rescue *Matn2* silencing, we modified the cDNA encoding full-length mouse Matn2 by mutating the sequence targeted by the shRNA but preserving its protein coding capacity (Fig. 4D). The modified cDNA was inserted into the pCEP-Pu expression vector and the recombinant plasmid was introduced into the sh3 cell line silenced for *Matn2*. Clones that were resistant to 1 µg/ml puromycin were pooled. Independent control (Ctrl), silenced (sh3, sh4, and sh7) cell lines and rescued (sh3-res2) cultures were tested for myogenic differentiation and marker gene expression in at least three independent experiments.

### Cell proliferation and differentiation assays

The MTS assay for mitochondrial function was performed with the CellTiter96 AQueous One solution cell proliferation kit (Promega), according to the manufacturer's protocol. For the evaluation of differentiation, myoblast cultures were stained for sarcomeric α-actinin and nuclei (DAPI). In randomly chosen microscope fields containing a total of 3000–4000 nuclei, α-actinin-positive myoblasts were counted. Primary myotube frequency was calculated as the number of myotubes containing <3 nuclei divided by the total number of cells in a given microscope field. The fusion index was defined as the number of myotubes harboring ≧3 nuclei divided by the total number of cells. The differentiation index was defined as the number of myotube nuclei divided by the total number of nuclei. Data represent the mean±s.e.m. from three to five independent experiments.

### Alternative promoter analysis

1-µl aliquots of total RNA samples treated with RNase-free DNase (Roche Molecular Biochemicals) were subjected to oligo(dT)-primed first-strand cDNA synthesis, followed by PCR using promoter-specific primer pairs (Fig. 6A), as described previously (Mátés et al., 2002).

### EMSA

The program Jaspar was used to search for putative NFI and MyoD recognition sequences in the Matn2 promoter and to synthesize double-stranded oligonucleotides (Fig. 6D). Constructs encoding GST–CTF1, an isoform of the human NFIC (Alevizopoulos et al., 1995) and GST–MyoD (Biesiada et al., 1999) were expressed, and the proteins were purified essentially as described previously (Nagy et al., 2011). 20–30 fmol of end-labeled wild-type and mutant DNA probes were incubated with 0.6–2.5 µg of GST or GST–MyoD in the presence of 300 ng of poly(dI-dC)•poly(dI-dC), as described previously (Biesiada et al., 1999). To test NFI binding, the probes were incubated with 1.25–2.5 µg of GST–CTF1 or with 1.25 µg each of GST-fused CTF1 and MyoD, as described previously (Szabó et al., 1995), and separated by electrophoresis on a pre-run 5% polyacrylamide gel.

### Transient expression assay

*Pd(−342)Luc* was constructed by inserting the −342/+60 P_d_ fragment into the *Pvu*II/*Pst*I site of the pGL3-Basic Vector (Promega). Derivatives carrying point mutations in the MyoD motif near the start site (Mdm1, Pd−12/+15) in the NFI motif (Nfm1) or in both the NFI and MyoD consensus sequences (NfMdm1) of the Nfi/MyoD composite element Pd−135/−107 were made using the PCR based QuikChange^TM^ Site-Directed Mutagenesis kit (Stratagene).

C2 myoblasts (cultured in growth medium) and COS-7 cells were transfected with 3–5 µg of wild-type and mutant reporters in six-well plates using ExGene 500 (Fermentas) or the calcium phosphate coprecipitation method, respectively, and luciferase activities were measured, as described previously (Nagy et al., 2011). Forced expression assays were performed with 3–5 µg of reporters and increasing amounts of expression plasmids for MyoD (pFMyoD, a gift from Vittorio Sartorelli) or mouse NFI proteins (pFNfia, pFNfib, pFNfic and pFNfix). The latter plasmids harbour the coding sequences of mouse NFI genes (Chaudhry et al., 1998) in pcDNA5′UT-FLAG, as described previously (Nagy et al., 2011). The effect of 50 ng/ml BMP7 (ImmunoTools) was tested in Opti-MEM® (Gibco). Transfection mixtures were adjusted with the appropriate empty vectors to the same amount of total DNA. Luciferase activities were expressed as fold values relative to that for *Pd(−342)Luc*. Results are the mean±s.e.m. from at least three independent experiments.

### Statistical analysis

Data are presented as the mean±s.e.m. Statistical significance was determined using a one-way analysis of variance (ANOVA) with KyPlot version 2.0 beta 15; *P<0.05, **P<0.01, ***P<0.001.

## Supplementary Material

Supplementary Material
