# Supplementary material for: Extracellular deposition of matrilin-2 controls the timing of the myogenic program during muscle regeneration
Source: J Cell Sci. 2014 Aug 1;127(15):3240–56. doi: 10.1242/jcs.141556 (PMC4117230; doi:10.1242/jcs.141556)
Supplement: Supplementary Material [file supp_127_15_3240__index.html]

Extracellular deposition of matrilin-2 controls the timing of the myogenic program during muscle regeneration — Supplementary Material 

# Extracellular deposition of matrilin-2 controls the timing of the myogenic program during muscle regeneration

## JCS141556 Supplementary Material

**Files in this Data Supplement:**

- **Supplementary Material**
