# Supplementary material for: Extracellular deposition of matrilin-2 controls the timing of the myogenic program during muscle regeneration
Source: J Cell Sci. 2014 Aug 1;127(15):3240–56. doi: 10.1242/jcs.141556 (PMC4117230; doi:10.1242/jcs.141556)
Supplement: Supplementary Material [file supp_127.15.3240_JCS141556.pdf]

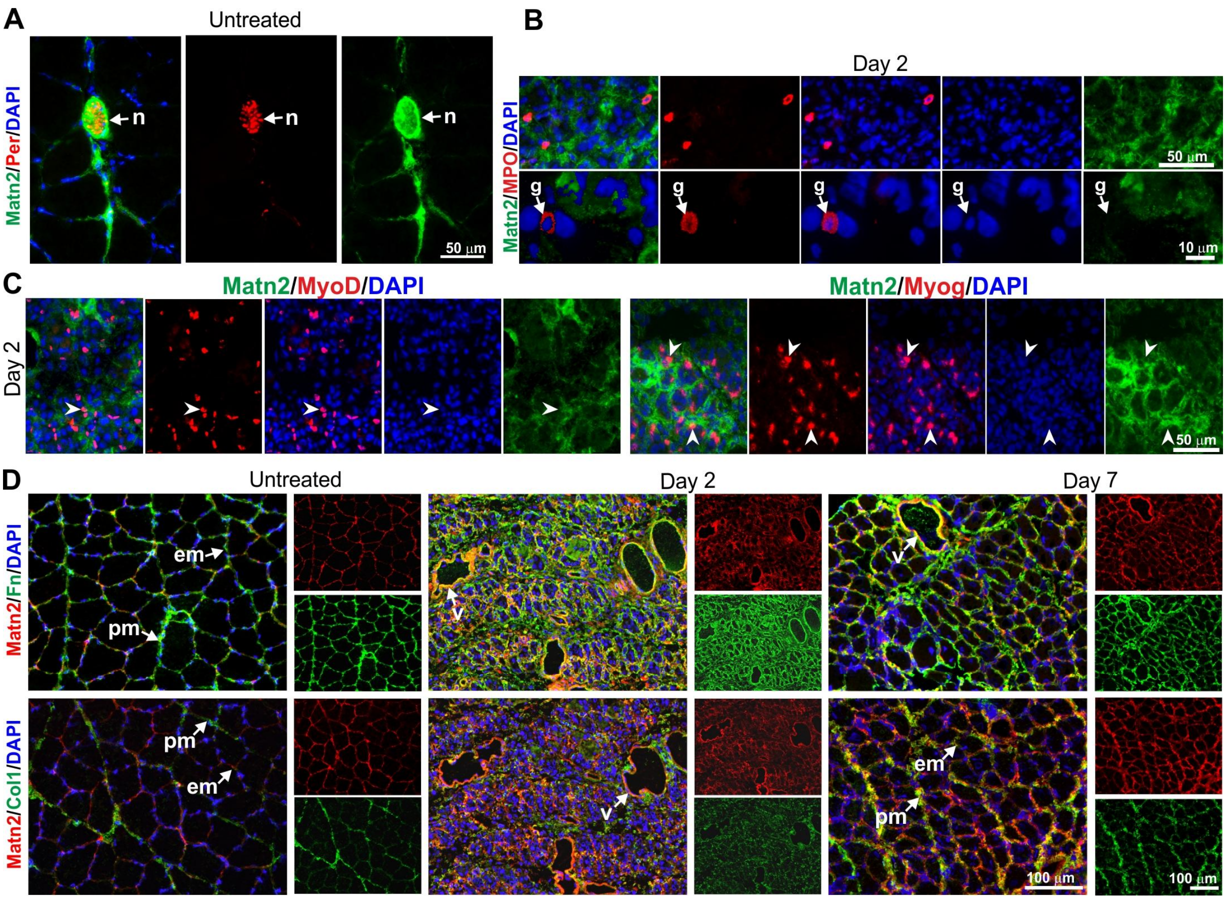

Fig. S1. Double immunofluorescence staining for Matn2 and other markers during rat soleus regeneration. (**A**) Strong Matn2 staining around nerve fibers visualized by peripherin (Per) staining in the untreated soleus. (**B**) Lack of Matn2 staining around granulocytes (g) expressing myeloperoxidase (MPO) two days postinjury. (**C**) Deposition of Matn2 around MyoD- and Myog-expressing cells (arrowhead) on day 2 of regeneration. (**D**) Matn2 shows partial colocalization with fibronectin (Fn) and collagen-1 (Col1) in the endomysium and perimysium of untreated and regenerating muscles. The colocalization of Matn2 and fibronectin is most evident on day 2 after treatment and around blood vessels. All three ECM proteins exhibit broader expression domain on days 2 and 7 of muscle regeneration compared to that of the untreated control muscle. Other symbols are as in Fig. 2.

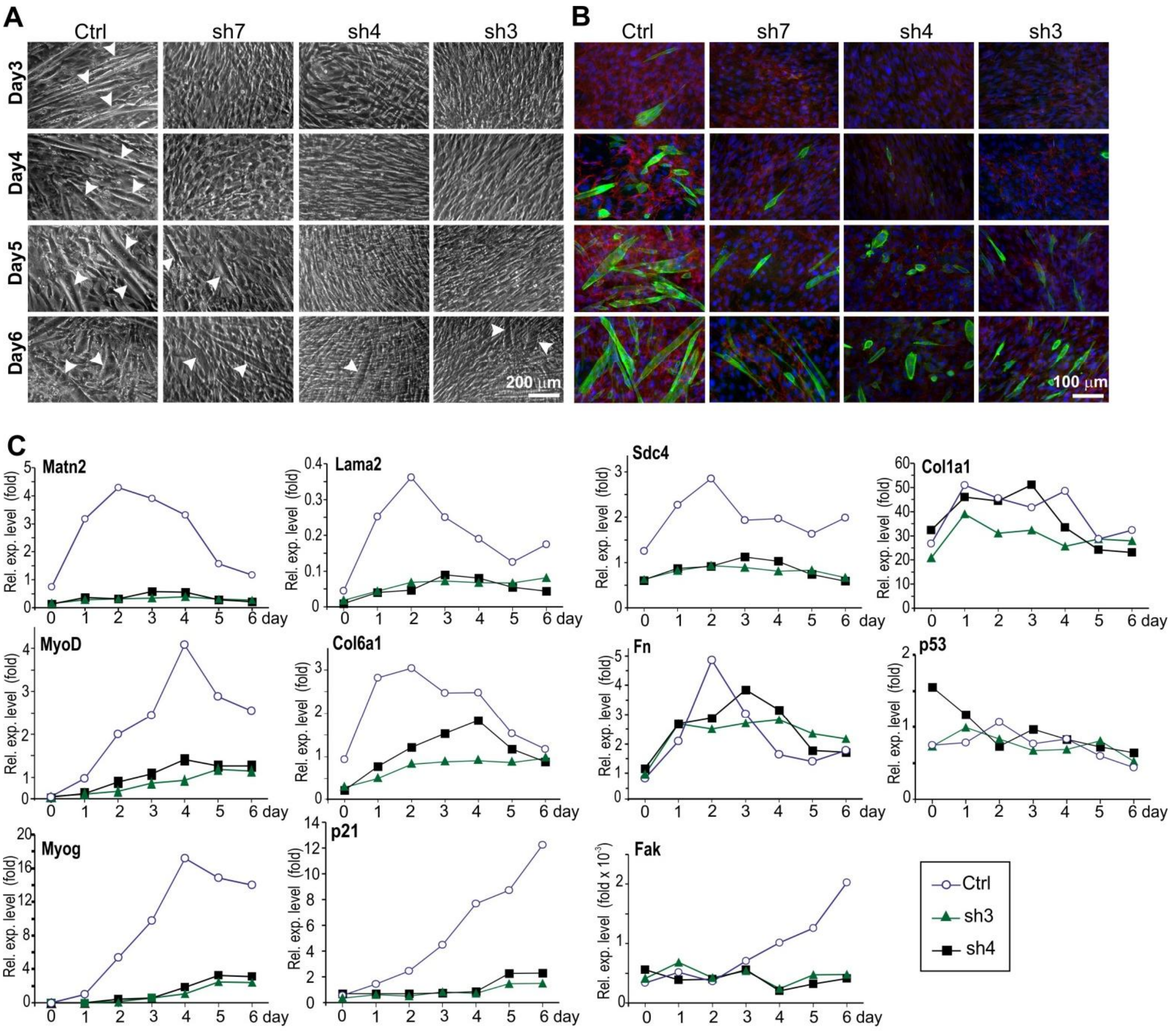

Fig. S2. Delayed myogenic differentiation and marker gene expression in various Matn2-silenced C2 cell lines. Comparison of phase-contrast microscopy (**A**), immunofluorescence (**B**) and marker mRNA levels (**C**) of parallel cultures of the Ctrl and silenced myoblast cell lines upon differentiation in DM. (A) Arrowheads point at multinucleated myotubes. (B) Double immunofluorescence staining of the cultures for Matn2 (red) and sarcomeric α-actinin (green). Note that the α-actinin-positive myotubes appear later, they are narrower and mononuclear in the *Matn2*-silenced cultures. (C) Marker gene expression measured by QRT-PCR analysis using the SYBR green protocol is plotted in fold relative to three internal control genes to illustrate reproducibility.

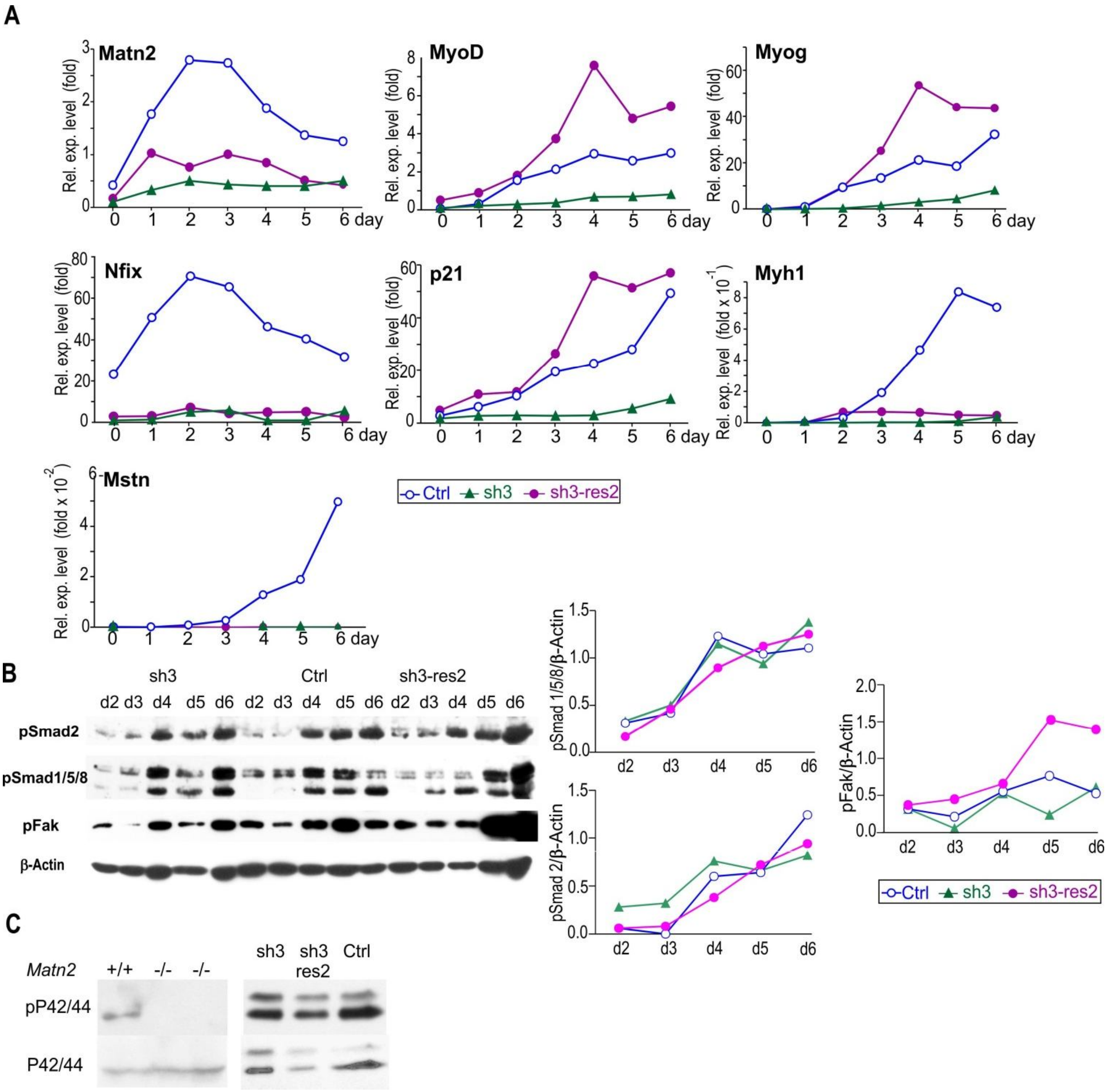

Fig. S3. **Marker gene expression changes in differentiating Matn2-silenced and rescued myoblasts cultures. (A)** Expression of *Nfix* and the early and late myogenic marker genes depends on the rescued Matn2 level. QRT-PCR analysis performed with TaqMan probes from another differentiation experiment of control, silenced and rescued myoblasts in DM is shown for comparison with Fig. 4I. Marker mRNA levels are presented as fold values relative to that of the HPRT mRNA. (**B**) Level of pSmads and pFAK in the sh3, Ctrl and rescued sh3-res2 myoblast cultures and quantification of the results. (**C**) Absence of *Matn2* affects P42/P44 phosphorylation in muscles of newborn mice (left panel) and in C2 myoblast cultures on day 2 of differentiation (right panel).

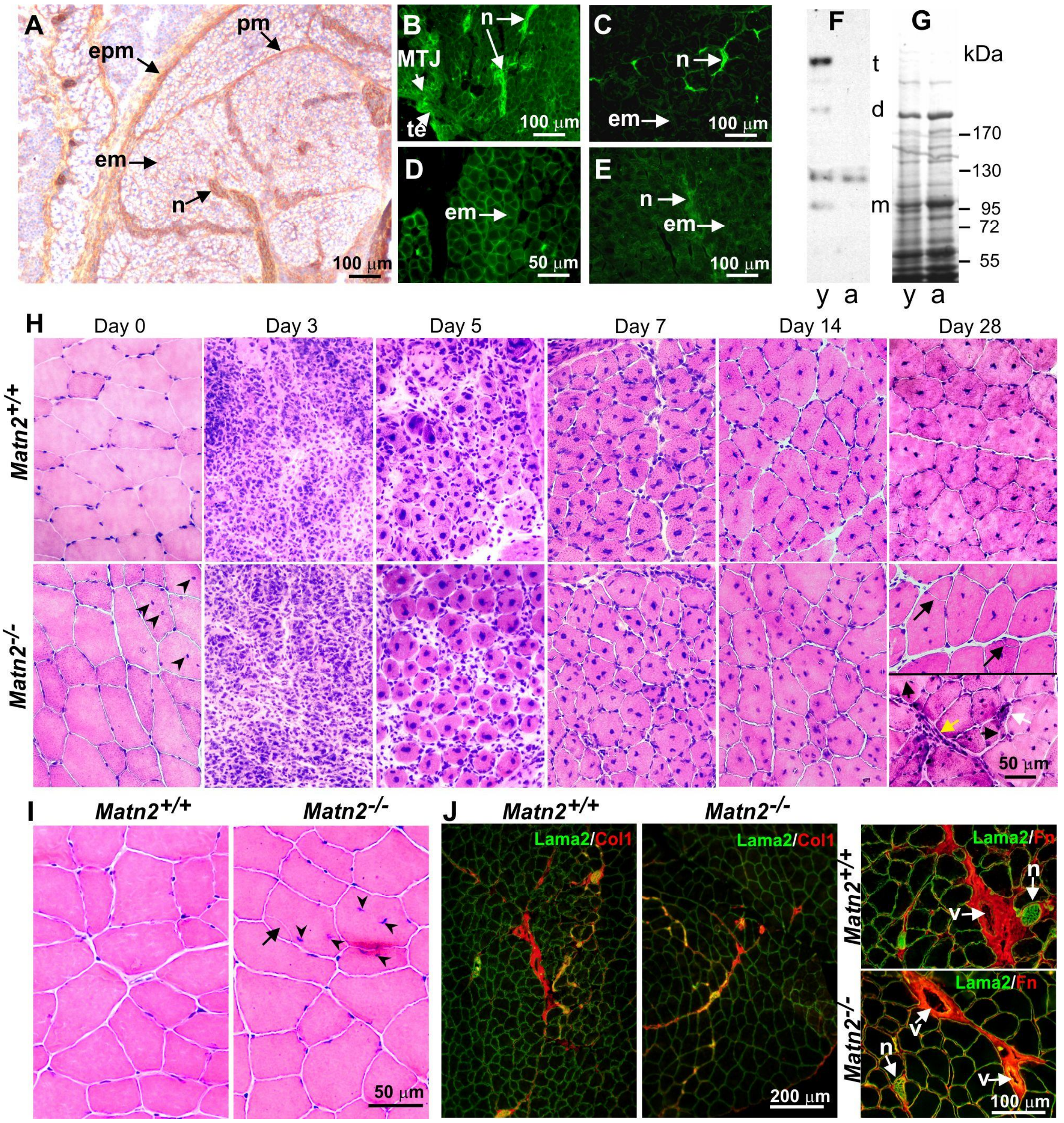

Fig. S4. Matn2 distribution in mouse skeletal muscle and notexin-induced TA regeneration. Matn2 immunohistochemistry (**A**) and immunofluorescence (**B-E**) in transverse cryosections of forelimb (A-C) and skull (D-E) muscles of newborn (A,B,D) and adult (C,E) mice. Deposition is seen in the epimysium (epm), perimysium, endomysium, nerves, MTJ and tendon (te). The signal intensities are moderate in the endomysium of muscle fibers in newborn animals and decreased further in adults. Immunoblot detection of Matn2 (**F**) and Coomassie Brilliant Blue staining (**G**) of muscle extracts of 15-day-old (y) and adult (a) mouse thigh. Without sample reduction, Matn2 is resolved predominantly as a trimer, but dimer, monomer and proteolytically processed forms are also visible in young mice (F). (**H,I**) H&E staining of TA cross sections from WT and *Matn2*$^{-/-}$ mice during regeneration (H) and at the age of 10 months (I). Arrowheads denote non-peripheral nuclei in untreated *Matn2*$^{-/-}$ mice, black arrows point to split fibers. In addition to fiber splitting, necrotic fibers with invading cells (white arrow) and increased ECM deposition (yellow

arrow) also occur in some areas of *Matn2*-deficient muscles on day 28 of regeneration (H). (**J**) Double staining of 10-month-old mouse TA shows collagen-1 and fibronectin deposition in connective tissues, especially around nerves and blood vessels, without signs of increased fibrosis in *Matn2*$^{-/-}$ mice. Other symbols are as in Figs. 1-3.

Table S1. Primary and secondary antibodies used in the study

Primary antibodies used in the study

Target	Species	Manufacturer or reference	Dilution for IF and IHC	Dilution for immunoblot
matrilin-2	rabbit	Piecha et al., 1999	1:100	1:3000
matrilin-2	goat	R&D, AF3234	1:200	
laminin	rabbit	DAKO, Z0097	1:400	
laminin α2	rabbit	Ringelmann et al., 1999	1:1000	
laminin α2	rat clone 4H8	Schuler and Sorokin, 1995	undiluted	
fibronectin	rabbit	Sigma, F3648	1:200	
Ki67	rat	DakoCytomation, M7249	1:100	
peripherin	rabbit	Chemicon	1:100	
MyoD1	mouse	Chemicon	1:30	
MyoD (C20)	rabbit	Santa Cruz, sc-304	1:50	
myogenin	mouse F5D	Thermo Scientific	1:30	
desmin	rabbit	Euro-Diagnostica	1:100	
desmin	mouse	DakoCytomation, M0760	1:200	1:1000
sarcomeric α-actinin,	mouse	SIGMA, A7811	1:400	
vinculin	mouse	SIGMA, V9131	1:400	
paxillin	mouse	SIGMA, P1093	1:400	
mouse collagen I,	rabbit	Millipore, AB765P	1:200	
collagen 3	goat	SouthernBiotech, 1330-01	1:200	
GFAP-Alexa488,	mouse GA5,	eBioscience 53-9892	1:500	
CD45	rat clone 30G12	Ralph and Berridge, 1984	undiluted	
myeloperoxidase	rabbit	NeoMarkers	1:30	
sarcomeric α-actin,	mouse	DakoCytomation, M0874		1:1000
Smad4	mouse	Santa Cruz, Sc-7966		1:1000
p21 (M-19)	rabbit	Santa Cruz, Sc-471		1:200
β-actin	mouse	SIGMA, A2228		1:5000
integrin a5	rabbit	Chemicon, AB1949		1:500
FAK (c-20)	rabbit	Santa Cruz, sc-558		1:1000
p-FAK (Tyr 397)	rabbit	Santa Cruz, sc-11765-R		1:1000
p-Smad2	rabbit	Invitrogen, 44-244G		1:1000
p-Smad1/5/8	rabbit	Cell Signaling Technology, 9511		1:1000
P44/42	rabbit	Cell Signaling Technology, 9102		1:1000
pP44/42	rabbit mAb	Cell Signaling Technology, 4370		1:1000

Secondary antibodies used in the study

Target	Species	Conjugated	Manufacturer	Dilution for IF and IHC	Dilution for immunoblot
rabbit IgG	donkey	Alexa488	Molecular Probes	1:500	
goat IgG	donkey	Cy3	Jackson Immunoresearch	1:400	
goat IgG	donkey	Cy2	Jackson Immunoresearch	1:400	
mouse IgG	donkey	Cy3	Jackson Immunoresearch	1:400	
mouse IgG	donkey	DyLight 488	Jackson Immunoresearch	1:100	
rat IgG	donkey	Cy3	Jackson Immunoresearch	1:400	
rabbit IgG	goat	HRP	Sigma, A6154	1:100	1:5000
mouse IgG	goat	HRP	Sigma, A9309		1:5000
goat IgG	rabbit	HRP	DakoCytomation, P0449		1:5000

Ralph SJ, Berridge MV (1984) J. Immunol. 132:2510-2514

Table S2. Primers used for amplification of hybridization probes and QRT-PCR

Symbol	Acc. No.	Forward sequence	Reverse sequence
Matn2 [a]	XM_216941	cctaccccaacggcataca	tgtgtgaacagtggcgaatc
MyoD [a]	NM_176079	ggttgagcgagaagcagga	gtagtaggcggcgtcgtag
Myog [a]	NM_017115	gacctgatggagctgtat	agacaatctcagttgggc
Gapdh [a]	NR_033630	ccagccccagcatcaaaggt	caactttggcatcgtggaag
Matn2 [b]	XM_216941	gaggtggagagggctgtcaag	gcaatgttgagggcatattgg
MyoD [b]	NM_176079	ggacagccggtgtgcatt	cactccggaaccccaacag
Myog [b]	NM_017115	ggagaagcgcaggctcaag	ttgagcagggtgctcctctt
Fak [b]	NM_013081	agtgacgtgtggatgtttgc	atccgttcttcttgctgcac
Matn2 [c]	NM_016762	gaggtggagagggctgtcaag	gcaatgttgagggcatattgg
Matn2 (ex1ex2) [c]	NM_016762	ctgctcccctgtctctcttc	cccaccaacatcttctccat
MyoD [c]	M84918	cgacaccgcctactacagtg	tatgctggacaggcagtcg
Myog [c]	NM_031189	ccttgctcagctccctca	tgggagttgcattcactgg
Myh1 [c]	NM_030679	aatcaaaggtcaaggcctacaa	gaatttggccaggttgacat
Trf3 [c]	AY457924	tcatctttagctctgggaaagtg	cagccgagactcctcttcac
Taf3 [c]	NM_027748	ctgccatcggtactctgaact	agttcatgcagattaactcccata
p21 [c]	NM_007669	tccacagcgatatccagaca	ggacatcaccaggattggac
Mstn [c]	NM_010834	tggccatgatcttgctgtaa	ccttgacttctaaaaagggattca
p53 [c]	NM_011640	acgcttctccgaagactgg	agggagctcgaggctgata
Lama2 [c]	NM_008481	tcaaccatgtgcttgtcctc	caatacacaagaggggctgaa
Col6a1 [c]	NM_009933	gacatccagggctccaaa	aggtgtcgagcacgaagaat
Sdc4 [c]	NM_011521	cccttccctgaagtgattga	agttccttgggctctgagg
Fn [c]	NM_010233	cggagagagtgcccctacta	cgatattggtgaatcgcaga
Col1a1 [c]	NM_007742	catgttcagctttgtggacct	gcagctgacttcagggatgt
Fak [c]	AB030035	agtgacgtgtggatgtttgc	atccgttcttcttgctgcac
Itga5 [c]	NM_008402	ggtgtggatcgagctgtctt	caaggccagcatttacagtg
Nfia [c]	NM_010905	ggaactcgatttatatttggcatac	ctggctgggactttcagatt
Nfib [c]	NM_008687	ccggaatacctggagtcg	gaaatggcaacggtgagg
Nfic [c]	NM_026756	catcgcggtacacagtgg	ggccgtatgggggaagta
Nfix [c]	NM_001081981	tgactcctccatcaccttca	gatccgatgctgacaaacc
Eno [c]	X62667.1	ctttgcacccaacatcctg	gcctggatggctgtctttag
Ckm [c]	NM_007710.2	cagcacagacagacactcagg	gaacttgttgtgggtgttgc
Rps18 [b, c]	NM_011296	tttgcgagtactcaacaccaa	ttcctcaacaccacatgagc
Hprt [b, c]	NM_013556	tcctcctcagaccgctttt	cctggttcatcatcgctaatc
CycloAb [b, c]	NM_008907	cacaaacggttcccagtttt	ttcccaaagaccacatgctt
Matn2	NM_016762.2	Mm01166023_m1 [d]	
MyoD1	NM_010866.2	Mm00440387_m1 [d]	
Myog	NM_031189.2	Mm00446194_m1 [d]	
p21	NM_007669.4	Mm01303209_m1 [d]	
Myh1	NM_030679.1	Mm01332489_m1 [d]	
Mstn	NM_010834.2	Mm00440328_m1 [d]	
Nfia	NM_001122952.1	Mm00447981_m1 [d]	
Nfix	NM_001081982.1	Mm00477796_m1 [d]	
Hprt	NM_013556.2	Mm00446968_m1 [d]	

[a] Primers used for amplification of cDNA probes in Northern hybridization

[b] Primers used in SYBR green protocol for rat samples

[c] Primers used in SYBR green protocol for mouse samples

[d] Applied Biosystems TaqMan probe sets
